# Addressing the Neglected Tropical Disease Podoconiosis in Northern Ethiopia: Lessons Learned from a New Community Podoconiosis Program

**DOI:** 10.1371/journal.pntd.0001560

**Published:** 2012-03-13

**Authors:** Sara Tomczyk, Abreham Tamiru, Gail Davey

**Affiliations:** 1 International Orthodox Christian Charities, Addis Ababa, Ethiopia; 2 International Orthodox Christian Charities, Debre Markos, Ethiopia; 3 Brighton and Sussex Medical School, Brighton, United Kingdom; Centers for Disease Control and Prevention, United States of America

## Abstract

**Background:**

Despite its great public health importance, few control initiatives addressing podoconiosis (non-filarial elephantiasis, a geochemical neglected tropical disease) exist. In June 2010, the first podoconiosis program in Northern Ethiopia, consisting of prevention, awareness, and care and support activities, began in Debre Markos, Northern Ethiopia. This study aims to document and disseminate the lessons learned from a new community podoconiosis program in Debre Markos.

**Methods/Principal Findings:**

We used a content analysis approach to examine and evaluate data from a series of sources. These sources include conducted interview transcripts, a focus group discussion transcript and secondary sources including monitoring and evaluation field reports, observation notes, and research obtained from a literature review. Themes were identified and grouped into matrix tables. Overall, sixteen program steps were identified and grouped into 6 domains: Initial preparation, training and sensitization, foundation building, treatment activity implementation, awareness, and follow-up. Emphasis is placed on the need for baseline data, effective training, local leadership, experience-sharing, mass-awareness, cross-cutting sector issues (i.e., water and waste management), and integration with government health systems. Related successes and challenges are also described, as are stakeholder roles and misconceptions and socio-cultural challenges affecting the program start-up. Many of the identified successes and challenges are relevant to the aim of the podoconiosis program to be sustainable and community-led.

**Conclusions/Significance:**

Much of this information has already been used to improve the Debre Markos program. We also anticipate that the domains and steps identified will be useful in guiding new programs in other settings where podoconiosis is highly prevalent. We hope to encourage partnerships and collaboration among podoconiosis stakeholders in future growth and disease control expansion.

## Author Summary

Podoconiosis is a chronic non-infectious disease that causes leg swelling among those living and walking bare-footed in red clay soil areas. It can be prevented and treated primarily by the use of shoes and foot hygiene. In Ethiopia, it is estimated that nearly 11 million people are at risk but few control programs exist. We aimed to assess and document the lessons learned from the first community podoconiosis program started in Northern Ethiopia in June 2010. We conducted interviews and a focus group discussion in addition to examining monitoring and evaluation field reports, observation notes, and other research articles. Overall, sixteen program steps were identified and grouped into 6 domains: Initial preparation, training and sensitization, foundation building, treatment activity implementation, awareness, and follow-up. Related successes and challenges, stakeholder roles, misconceptions and socio-cultural challenges affecting the program start-up were also identified. We hope that the results will be useful in guiding new programs in other settings where podoconiosis is highly prevalent.

## Introduction

Podoconiosis, or non-filarial elephantiasis, is a geochemical type of neglected tropical disease that affects individuals, often barefoot subsistence farmers, exposed to the red clay soil originating from volcanic rock [1]. Irritant particles in this soil penetrate the skin of the foot resulting in a progressive obliterative endolymphangitis. Although further studies are needed to fully understand the pathogenesis of podoconiosis, it has been demonstrated that colloid-sized particles of common irritant clay elements such as aluminum, silicon, magnesium and iron are present in lower limb lymph node macrophages of affected and non-affected individuals living in podoconiosis endemic regions. Evidence suggests that for those that are genetically susceptible, the primary lymphatic vessels become dilated, and edema and disorganized collagen production occurs, eventually obliterating the lymphatic vessel lumen [2]. Clinically, this causes debilitating lymphoedema of the lower leg, with or without skin changes, including hyperkeratosis, ‘mossy’ papillomata, and fibrotic nodule formation [3]. Early symptoms include itching of the forefoot skin, burning sensations in the foot and lower leg, splaying of the forefoot, plantar edema, and increased skin markings. On average, affected patients also experience 5 acute episodes (acute adenolymphangitis, ALA) per year, consisting of pyrexia, a warm, painful limb, and possible progression to a harder, fibrotic leg [2].

Podoconiosis is staged using an adapted Dreyer staging system for lymphatic filariasis. This adapted system has five stages based on the proximal spread of swelling, knobs and bumps in addition to the measurement of moss presence (M+ or M−) and the below-knee circumference. Podoconiosis can be distinguished from filarial elephantiasis through history and clinical examination: podoconiosis develops first in the foot, it causes bilateral but asymmetric swelling often confined to the lower leg, and groin involvement is rare in podoconiosis. In contrast, lymphatic filariasis can extend above the knee and has the potential for groin involvement. Another common differential diagnosis apart from podoconiosis is leprosy lymphedema. Patients with podoconiosis can be distinguished from leprosy lymphedema because sensation persists in the toes and forefoot, and trophic ulcers, thickened nerves and hand involvement are not experienced [2]. In endemic areas, podoconiosis has shown to be reliably diagnosed by lay health workers [4].

Podoconiosis is of public health importance in highland zones of Africa [5]. In Ethiopia alone, nearly 11 million people or approximately 18% of the population are at risk of podoconiosis [6]. In a podoconiosis endemic zone in Southern Ethiopia, a cross-sectional survey estimated a podoconiosis prevalence of 5.46% [7]. Another cross-sectional survey conducted in Western Ethiopia found 2.8% disease prevalence. These figures projected to the population living on irritant soil throughout the country suggest that approximately one million Ethiopians may be affected by the disease [8]. Podoconiosis is also associated with significant economic and social burdens. In a study by Tekola et. al, it was found that affected individuals lose 45% of their total productive work days, costing a single zone of 1.5 million people in Ethiopia more than 16 million USD annually [9]. Furthermore, another cross-sectional study in Southern Ethiopia found that more than one-half of respondents studied showed stigmatizing attitudes towards social interactions with podoconiosis patients [10].

Despite the public health importance and prevalence of podoconiosis, few control initiatives exist. In Wolayta, Southern Ethiopia, the Mossy Foot Treatment and Prevention Association (MFTPA) has been recognized as a successful ongoing podoconiosis community program model since 1998 as compared to the World Health Organization (WHO) Innovative Care for Chronic Conditions (ICCC) Framework [11]. The MFTPA program consists of prevention (distribution of shoes to children, adult shoemaking), treatment (hygiene/shoe wearing education integrated into clinics), and rehabilitation (microcredit, training) activities at the community-level across one zone. In June 2010, the first podoconiosis program in Northern Ethiopia was started in Debre Markos, East Gojam Zone in an effort to take the experiences of MFTPA and develop a program specific to the context of Northern Ethiopia. The program in Debre Markos aims to address podoconiosis prevention, awareness, and care and support activities. We report a qualitative evaluation of this community-based health program designed to document and disseminate the lessons learned from its launch in a region that had yet to see podoconiosis interventions. In particular, we identify the processes the project underwent including the relevant challenges and successes, the stakeholder roles, and the misconceptions and socio-cultural factors affecting program implementation.

## Methods

### Ethical Considerations

This evaluation was performed to provide feedback to the Charities & Societies Agency, local stakeholders and funders, as required by non-government organizations under Ethiopian law. The evaluation was not submitted to an IRB or ethical committee review because information was gathered with the above purpose in mind, and no additional data including individual patient medical records were gathered purely for research purposes. Oral consent was obtained from participants prior to interviews and documented in a participant checklist. Oral consent was used instead of written consent because a portion of the participants were illiterate.

### Study Population

The study was conducted in East Gojam Zone, Amhara Region, Northern Ethiopia. The zonal capital is Debre Markos, a town located 300 km northwest of the capital city Addis Ababa at an altitude of 2446 meters in the highlands above the Blue Nile Gorge. The main spoken language is Amharic [12]. The population of East Gojam Zone is 2,171,998 people. An estimated 89% of this population relies on subsistence farming as a source of income. The rainfall in East Gojam Zone is 1200 to 1500 mm per annum [13]. Podoconiosis prevalence has not been measured in the area.

### The Podoconiosis Program

This study focused on the first year of the new Debre Markos community podoconiosis program (see Figure S1). This program is led by one coordinator and support staff including one nurse, six shoemakers, and four volunteers given stipends. In the pilot phase, 150 patients were enrolled into the program and the remaining patients requesting treatment were registered on a waiting list. The program consists of prevention, awareness, and treatment activities. In particular, a simple lymphoedema treatment was emphasized, including foot hygiene through daily washing with soap and water, diluted bleach soaks as an antiseptic, the regular use of an emollient, elevation at night, the use of shoes, and attention to additional needs such as wound care, acute attacks, and psychological support [3]. Prevention activities include school education, and awareness is achieved through community-at-large awareness events and materials such as brochures and t-shirts with podoconiosis education messages.

### Study Design

Data were collected through a series of eight semi-structured interviews (5 males, 3 females; average age 27.3; age range from 22 to 36) and one focus group discussion (5 males, 6 females; average age 37.8; age range from 26 to 49). The interviewees were selected to represent stakeholders involved in the set-up of the program, including community members, health professionals, governmental officials, church leaders, and program staff. Stakeholders can be defined as “actors who have an interest in the issue under consideration, who are affected by the issue, or who because of their position have or could have an active or passive influence on the decision-making and implementation process,” [14]. The focus group discussion (FGD) participants consisted of program beneficiaries. All interviewees and FGD participants provided informed consent prior to the dialogue and the objective of the evaluation was described. The FGD was facilitated by hosting it in the same treatment environment that program beneficiaries were accustomed to. Face-to-face open-ended questions were used to gain comprehensive qualitative information and responses were recorded in hand-written notes. These notes were then compiled in a typed transcript. Two of the interviews were conducted in English and the remaining 6 interviews and the FGD were conducted in Amharic (the National Ethiopian language.) Additional information was collected through secondary sources such as monitoring and evaluation reports, observation notes, and a literature review.

### Analysis

A content analysis approach was used to examine the interview transcripts, the FGD transcript, the monitoring and evaluation field reports, observation notes, and literature. Each document was examined jointly by two of the investigators multiple times. In order to minimize interviewer biases, the interview/FGD transcripts were made anonymous during analysis. Phrases relating to program start-up steps, stakeholder roles, and misconceptions and socio-cultural challenges affecting the new program were identified and grouped into themes and matrix tables. These themes were continuously reviewed and changed as new ideas arose to ensure completeness.

## Results

From the data collected, sixteen program steps were identified including relevant program successes and challenges. These steps were grouped into 6 domains: Initial preparation, training and sensitization, foundation building, treatment activities implementation, awareness, and follow-up. As seen in *Tables 1* *–* [Table pntd-0001560-t002] [Table pntd-0001560-t003] [Table pntd-0001560-t004] [Table pntd-0001560-t005] *6*, the first initial preparation domain includes the following three steps: Gather data on endemicity to begin identifying implementation areas; Identify local stakeholders and engage in discussion to further identify a program implementation area; Approach the local government and community leaders in the identified area. (Sources: Interviews, FGD, monitoring and evaluation reports, observation notes.) The second training and sensitization domain includes the following four steps: Organize a one day program sensitization workshop; Identify program support staff; Program coordinator and nurse attend training at established program; Organize a half day training on how to conduct a baseline assessment. (Sources: Interviews, monitoring and evaluation reports, observation notes.) The third foundation building domain consists of the following three steps: conduct a baseline assessment or household survey (462 podoconiosis affected individuals were identified from four villages with an estimated total population of 12,650, giving an approximate disease prevalence of 3.7%); Identify a treatment site; Purchase treatment and shoemaking supplies. (Sources: Interviews, FGD, monitoring and evaluation reports, observation notes.) The fourth treatment activity implementation domain consists of two steps: Arrange for an experienced podoconiosis program shoemaker to provide training and begin shoemaking; Begin weekly treatment meetings. (Sources: Interviews, FGD, monitoring and evaluation reports, observation notes.) The fifth awareness domain includes two steps: Develop Information Education Communication/Behavioral Change Communication (IEC/BCC) materials; Host podoconiosis community-at-large awareness events. (Sources: Interviews, FGD, monitoring and evaluation reports, observation notes.) Finally, the sixth follow-up domain consists of two steps: Follow-up with patients at household level; Develop monitoring and evaluation (M&E) forms. (Sources: Interviews, monitoring and evaluation reports, observation notes, literature review.)

**Table 1:** Domain One: Initial preparation.

*Steps*	*Notes*	*Successes*	*Challenges*
A. Gather data on endemicity to begin identifying implementation areas	These data arose from documented observation.		
B. Identify local stakeholders and engage in discussion to further identify a program implementation area.	A local stakeholder was identified as a possible local coordinator based on demonstrated interest and capability in relevant public health areas.	The local coordinator had established relationships with the government, health system and community which provided momentum.	
C. Approach the local government and community leaders in the identified area	The government was allowed to take leadership and ownership was encouraged.		

**Table 2 pntd-0001560-t002:** Domain Two: Training and sensitization.

*Steps*	*Notes*	*Successes*	*Challenges*
A. Organize a one day program sensitization workshop	The workshop was led by a disease expert and included demonstration and active participation.		A strategic plan was not prepared during this workshop. It should have been discussed before the beginning of the program to facilitate more government and community ownership and identify gaps.
B. Identify program support staff	Program support staff was identified based on experience, initiative demonstrated during the program sensitization workshop and recommended by the local coordinator.		It was difficult to integrate treated patients as program support staff because it was a new program area.
C. Program coordinator and nurse attend training at established program	This program was MFTPA in southern Ethiopia.	This exposure was essential for generating understanding and commitment.	
D. Organize a half day training on how to conduct a baseline assessment	Field workers showing initiative were identified from the program sensitization workshop.		

**Table 3 pntd-0001560-t003:** Domain Three: Foundation building.

*Steps*	*Notes*	*Successes*	*Challenges*
A. Conduct a baseline assessment or household survey	Areas were selected based on observed high burden of disease and government input. The survey was conducted by field workers(1 volunteer covered approx. 200 households in 5 days). The survey included demographics, clinical staging, shoe sizing, etc. From four villages with an estimated total population of 12,650, 462 podoconiosis cases were identified for an approximate disease prevalence of 3.7%.	This household to household assessment was also a means to mobilize patients. Patients were registered and appointed to a first meeting date. Patients were additionally found through kebele (administrative unit) leaders.	The identified disease prevalence from the Debre Markos baseline assessment was an estimate. Limited supervision affected data collection quality.
B. Identify a treatment site	Patients were appointed during the baseline assessment to a general community gathering spot for registration and program introduction and the regular program site was then identified based on the baseline assessment results and presence of the following items: access to water, liquid waste management capacity, and space for privacy and comfort.	The local government contributed space for the treatment site within the compound of a government health clinic, promoting government ownership and mainstreaming.	The initial site was unsuitable because of lack of water, crowding, lack of liquid waste management, and lack of office space for supplies/documentation.
C. Purchase treatment and shoemaking supplies	Most treatment supplies were purchased from the local market and the shoemaking supplies from the capital city or larger market.		

**Table 4 pntd-0001560-t004:** Domain Four: Treatment activity implementation.

*Steps*	*Notes*	*Successes*	*Challenges*
A. Arrange for an experienced podoconiosis program shoemaker to provide training and begin shoemaking	An experienced shoemaker traveled to the new program site to provide the training.	Shoemaking was located on the treatment site and this brought meaning and commitment to the shoemakers' work. The use of shoes improves bandage use. Shoemaking built skills and provided income for the shoemakers.	The shoemaking component took time to start. Shoemaking should have been organized earlier because of the difference shoes make in treatment progress. Socks should be distributed at the same time as shoes to prevent exposure to the soil and painful friction with wounds.
B. Begin weekly treatment meetings	These meetings included patient registration, clinical staging, photo documentation, measurement of shoe size, health education, distribution of hygiene supplies, foot hygiene demonstration and practice, and psychological support. A subgroup of patients needed bandages, antibiotics for acute attacks, or wound care. Shoes were made and distributed based on need. Treatment meetings were held weekly in the beginning and subsequent frequency can be determined based on performance/needs.	Patients paid subsidized fees for hygiene supplies (2 ETB = 0.12 USD) and shoes (10 ETB = 0.60USD) which increased patient ownership. Committed returning patients were asked to share their treatment experience and help other patients, which encouraged patient motivation and behavioral change. Patient progress and clinical staging was tracked through registers and photo documentation which promoted accurate case management and patient motivation. Patients reported validation and improved mental health from individual and group counseling and the private space of the treatment site. Program staff reported mental and spiritual growth/satisfaction from their participation in the program. Patients were asked “What is your contribution?” (i.e. mobilizing other patients, spreading education/awareness messages, etc.) which furthered ownership.	Significantly more patients than anticipated arrived at the first treatment meeting creating a chaotic environment. It was more appropriate to focus the first meeting on registration and then assign patients into small groups (each approximately 30 patients) with each group meeting on a different day of the week. More patients than available resources approached the program creating a demand versus capacity challenge. A waiting list was created and those on the list were provided oral health education and then appointed to a date the next month to check availability. Hygiene education was addressed first, whereas it took time to start addressing acute attacks. Acute attacks must be dealt with immediately since they are painful and linked to disease progression.

**Table 5 pntd-0001560-t005:** Domain Five: Awareness.

*Steps*	*Notes*	*Successes*	*Challenges*
A. Develop Information Education Communication/Behavioral Change Communication (IEC/BCC) materials	T-shirts with education messages were used as incentives. Brochures in the local language and in English were distributed to multi-level stakeholders including at the government and community level in addition to the international community.		
B. Host podoconiosis community-at-large awareness events.	“Community conversations” or social community meetings were held (coffee and tea were offered; and podoconiosis was discussed among the group.	Patients were asked to present and share their stories at these community events to increase their effectiveness and increase the self-esteem of the patients. After providing podoconiosis education, community members were asked to talk about the disease and suggest methods of prevention or ways to prevent stigma. Community leaders were asked to endorse these events to increase further ownership.	

**Table 6:** Domain Six: Follow-up.

*Steps*	*Notes*	*Successes*	*Challenges*
A. Follow-up with patients at household level	The health volunteers traveled house to house to those too ill to attend for treatment or with poor treatment adherence		
B. Develop monitoring and evaluation (M&E) forms.	These forms were created with local input which may necessitate several drafts.	These forms were used to track indicators, challenges, success stories, etc.	

Additional notes, successes, and challenges are documented for each step. Emphasis is placed on the need to gather baseline data, for effective training, local leadership, experience-sharing, mass-awareness, cross-cutting sector issues (i.e. water and waste management), and integration with government health systems. Related successes and challenges were also identified, many of which are relevant to the aim of the podoconiosis program to be sustainable and community-led.

Across these program steps, many different stakeholders were found to hold a role, each being significant to the success of the activities. Stakeholder roles in establishing the new program were identified as shown in *Table 7* *.*

**Table 7:** Stakeholder roles in the start-up of the program.

Stakeholder	Role
1. Government	Recognize podoconiosis social, cultural, and economic disease burden and advocate appropriately; Oversee overall coordination and technical support to existing podoconiosis programs; Integrate podoconiosis prevention and treatment with existing health activities; Mobilize society to commit materials/labor to podoconiosis program infrastructure; Contribute podoconiosis treatment site (i.e. on government clinic compound); Promote podoconiosis awareness and stigma prevention opportunities through community leaders, politic gatherings, public events, village census, etc.; Ensure compliance with national health policies and plans
2. Health Professionals	Recognize the podoconiosis disease burden, etiology, and impact it has on life; Provide appropriate podoconiosis treatment and education
3. Non-Governmental Organizations (NGOs)	Provide financial and coordination support to podoconiosis activities; Identify interested and empowered local professionals as podoconiosis program staff; Report on podoconiosis program indicators and progress; Ensure regular podoconiosis program staff supervision
4. Podoconiosis Affected Individuals	Practice podoconiosis treatment adherence; Mobilize and educate other affected individuals; After training, lead local treatment groups; Perform local advocacy
5. Community	Follow good podoconiosis prevention practices; Contribute materials/labor to program infrastructure; Educate and mobilize affected individuals; Advocate against stigma
6. Faith-Based Institutions	Religious leaders provide sermons integrating podoconiosis health messages; Educate and mobilize affected individuals
7. Schools	Educate children on podoconiosis awareness and prevention practices; Encourage children to educate other family members

A range of misconceptions about the disease and about wearing shoes were reported by community members and affected individuals, many of which are likely to influence the program in its early stages.

### Beliefs about podoconiosis

A wide range of beliefs about podoconiosis were expressed, ranging from physical to spiritual –

“Some people believe that podoconiosis is caused from standing up too fast or the hot soil burning their skin” (Male church member, 31 years.)

“The community doesn't believe that the cause of podoconiosis comes from the soil. More people think that it comes from contact with blood, evil spirits, or a curse from God” (Male health worker, 29 years.)

“Many relate podoconiosis to magic” (Male shoemaker, 23 years.)

“Some believe podoconiosis is caused by wearing bad shoes or acts of sin by individuals or families” (Male city worker, 28 years.)

“We thought podoconiosis was caused by evil spirits, family links, sunlight, or touching the soil around cemetery graves (Female house-keeper, 28 years.)

“Some of us just accepted that we had to live with an unknown condition with an unknown cause” (Female farmer, 47 years.)

Others think podoconiosis comes from contact with animal blood. These beliefs affect the program because it takes time to convince patients otherwise” (Female nurse, 22 years old.)

### Beliefs about shoes and shoe wearing

“Many believe that shoes are a sign of laziness. The sign of strength is going without shoes. (Female nurse, 22 years old.)

### Challenges faced by people with podoconiosis

Affected individuals and community members also reported many challenges faced by people with podoconiosis in daily life. Several of these are likely to have direct impact on attendance at a newly launched program.

“Many persons wade in the river to be able to “wash” their feet but the river is thick with silica soil and it doesn't properly clean their feet. Others are too tired to wash their feet after a long day working in the fields.” (Male health worker, 31 years.)

“Religious practices affect the hygiene necessary to prevent podoconiosis. For example, after communion, Orthodox lay persons often don't wash their body for one day. Once affected, podoconiosis patients then can become excommunicated because of stigma. Women affected by podoconiosis may not be able to marry” (Male church member, 31 years.)

“Everyone stigmatizes patients because they are not with God” (Male health worker, 29 years.)

“Patients mostly think that if they are seen going for treatment, they will be further stigmatized and they don't believe there is hope” (Male city worker, 31 years.)

“Many of us patients aren't registered because it is too difficult to travel from remote areas” (Male farmer, 31 years.)

“Patients' use of traditional medicine and inability to buy shoes in addition to a lack of attention by clinical health workers negatively affect treatment health programs” (Male city worker, 28 years.)

## Discussion

The results gathered are intended to report the lessons learned from the start-up of the Debre Markos podoconiosis program, the first of its kind in Northern Ethiopia. From these results, two important lessons learned can be noted as critical components to effective program start-up:

- the importance of government engagement through a training workshop and joint development of a strategic plan, and
- the need for early community events and engagement aimed at diminishing stigma and fatalism, enabling patients to identify themselves and come forward for treatment.

The focus group discussion of patients with podoconiosis added particular value to the results presented. Their experiences approaching and navigating the podoconiosis treatment for the first time and their experiences with associated stigma provided a unique perspective that also dictates local podoconiosis program start-up steps. In addition, the misconceptions concerning podoconiosis and the socio-cultural challenges reported by people with podoconiosis were likely to influence a new program.

“These beliefs affect the program because it takes time to convince patients otherwise” (Female nurse, 22 years old.)

It was our experience that these misconceptions act as barriers to new programs because patients need to be educated on the true cause of disease and convinced of the value of the hygiene treatment practices suggested. The practical clinical improvement seen by patients was a powerful tool in overcoming these misconceptions. After witnessing the improvement of others with the disease, a majority of patients turned away from their earlier misconceptions about the cause of podoconiosis and were more willing to follow the recommended hygiene treatment. The daily challenges faced by people with podoconiosis also create additional program barriers that need to be addressed in a sensitive manner.

Overall, we hope the results of this study are a fair and honest representation of the perceptions, challenges, and successes surrounding the beginning of the Debre Markos program and its ability to address podoconiosis. However, several limitations should be noted in this study's approach.

An “insider-outsider” perspective was taken to conduct the study, meaning that the authors were involved in the events described in the study [15]. There is a risk that the perspective of the authors comes across too strongly a voice in the results of the study or that ‘social desirability’ biases existed in the case of reporting program challenges or mistakes. Furthermore, the authors were present at a number of the semi-structured interviews and focus group discussion, so there is a clear possibility for reporting bias. In contrast, the “insider-outsider” perspective may add strength to telling the story of the Debre Markos podoconiosis program by combining the words of the authors and a range of different informants. Regardless, no attempt is made here to state that the insights are neutral or not influenced by the involvement of the authors. Additionally, the study moved between international and national staff which may have resulted in subtle cultural and interpersonal communication constraints which also could have affected the study outcomes.

This “insider-outsider” perspective captured a cross-section of stakeholders. It could have been colored by changing positions, events, and context. The study was conducted as an evaluation to provide feedback to the Charities & Societies Agency and local stakeholders and was thus, resource-constrained. If further resources had been available, the evaluation could have been made more comprehensive by including an expanded cross-section of stakeholders and additional community participatory methods. Particular stakeholders such as those from the education sector were not included in the interviews. As noted in *Table 2*, the school plays an important role in disease prevention, awareness, and stigma and should hold weight in the start-up of community podoconiosis programs. In all program steps identified, government ownership emerges as an important factor. More reflection at government level may strengthen application of the lessons identified at the start of future podoconiosis programs. Additionally, the conducted baseline assessment which showed results of a 3.7% prevalence of podoconiosis in the area could be inaccurate estimate as limited supervision and lack of a questionnaire pre-test may have affected data collection quality.

It should also be recognized that other groups such as the Mossy Foot Treatment and Prevention Association (MFTPA) in Wolayta, Southern Ethiopia have important experience to contribute to other podoconiosis programs. Against the WHO ICCC Framework, it was found that their strength at the micro-level is evident particularly through involvement of treated patients as community patient agents (CPAs) and through network groups composed of local leaders [10]. Their experience in clinical, social work, and administration areas relevant to podoconiosis may be considered ‘best practice.’ The ICCC Framework consists of micro (i.e. patient), meso (organization/community), and macro (policy) levels and was created by a WHO working group to enable health care systems to design programs to more effectively manage long-term health problems [11]. In addition, there are different lymphedema management programs in Africa and India on basic lymphedema hygiene self-care for individuals affected with lymphatic filariasis that could provide other clinical support models [16].

This study was conducted to document and disseminate the lessons learned from the experience of starting up a community-based prevention program. These lessons are illustrated in the form of project process steps, related project challenges and successes, stakeholder roles, and misconceptions and socio-cultural factors affecting program start-up. The results of the analysis have been used to improve the design and implementation of the present Debre Markos program and increase its chances of sustainability. Logical frameworks have been developed and assumptions have been identified. This qualitative information could hold more depth than quantitative survey research in designing strategies for change.

We anticipate that these lessons will be useful in guiding future programs in other settings where podoconiosis is highly prevalent. In the future, a comprehensive regional and national podoconiosis prevalence mapping is necessary to identify endemic areas with high disease burden. Associated patient-led groups should be established in more remote areas identified to further the reach of the treatment site and an effective patient ‘graduation’ scheme needs development. Government ownership must continue to be promoted at all national, regional, and local levels. Podoconiosis activities should be mainstreamed through the appropriate government directives (i.e. directives encouraging local health bodies to take leadership) and decentralized community initiatives. Specific pre-service and in-service training for health professionals at every level is necessary. Partnerships with local government bodies and other existing NGOs must be encouraged and podoconiosis prevention and treatment activities integrated with existing school health education programs and community water projects. Currently, there are school health education programs implemented in Amhara Region that focus on hygiene education through peer-group leadership and creative activities such as music and drama, which might be used for podoconiosis education. The following domain steps might also provide opportunities for integration with other neglected tropical diseases: Domain One. Initial Preparation/Step C. Approach the local government and community leaders in the identified area (government ownership); Domain Two. Training and Sensitization/Step A. Organize a one day program sensitization workshop and Domain Five. Awareness/Step A. Develop Information Education Communication/Behavioural Change Communication materials and Domain Six. Follow-up/Step A. Follow-up with patients at household level. There is a common hygiene message with lymphatic filariasis (however, it should be noted that podoconiosis is endemic in regions at 1000 m–2000 m above sea level where lymphatic filariasis is not typically found), trachoma, soil-transmitted helminthes and a common shoe use message with soil-transmitted helminthes [2]. Additionally, integration and partnership opportunities could also be considered with conditions related to “integrated limb care”, such as diabetes, venous insufficiency, and the concerned neglected tropical diseases.

With this study, we also hope to encourage partnerships and collaboration among podoconiosis stakeholders. The potential for experience-sharing and collaboration among podoconiosis stakeholders has the power to arouse public interest and stimulate action in addressing this neglected tropical disease.

## Supporting Information

Figure S1 **Debre Markos podoconiosis program (began 2010) logframe with vision, goal, activities, and monthly measurable indicators.**
(DOC)
