# Supplementary material for: Addressing the Neglected Tropical Disease Podoconiosis in Northern Ethiopia: Lessons Learned from a New Community Podoconiosis Program
Source: PLoS Negl Trop Dis. 2012 Mar 13;6(3):e1560. doi: 10.1371/journal.pntd.0001560 (PMC3302806; doi:10.1371/journal.pntd.0001560)
Supplement: Figure S1 — Debre Markos podoconiosis program (began 2010) logframe with vision, goal, activities, and monthly measurable indicators. (DOC) [file pntd.0001560.s001.doc]

**Debre Markos Podoconiosis Program (Began 2010)**

**Logframe**

| **Aims** | **Monthly Measurable Indicators** |
| --- | --- |
| **Vision:**  To control podoconiosis in Northern Ethiopia. |  |
| **Goal:**  To provided podoconiosis care and support in Debre Markos, a podoconiosis endemic area in Amhara Region, Northern Ethiopia. |  |
| **Activities:**  1. Treatment: Provide hygiene activities | Number of individuals attending weekly treatment meetings.  Number of individuals showing decreased leg circumference.  Number of individuals showing decreased moss.  Number of individuals with decreased clinical stage.  Number of individuals being seen on home visits.  Number of individuals that received treatment hygiene supplies. |
| 2. Treatment: Organize shoemaking activities and distribution of completed shoes and socks | Number of shoes produced.  Number of people that received program shoes and socks. |
| 3. Treatment: Provide wound care and acute attack care activities | Number of individuals with wounds.  Number of individuals with acute attacks.  Number of individuals that received medication to address infected wounds.  Number of individuals that received medication to address acute attacks. |
| 4. Awareness: Organize community social gatherings with podoconiosis presentations | Number of individuals attending social gatherings with podoconiosis presentations.  Number of podoconiosis education materials (IEC/BCC) distributed. |
| 5. Prevention: Organize distribution of shoes to children at schools through a held podoconiosis education activity | Number of children that received shoes.  Number of individuals (teachers, children and family members) attending podoconiosis education activity. |
